# A retrospective analysis of clinical features of patients hospitalized with SARS-CoV-2 Omicron variants BA.1 and BA.2

**DOI:** 10.1038/s41598-023-34712-9

**Published:** 2023-05-16

**Authors:** Cristina Groza, David Totschnig, Christoph Wenisch, Johanna Atamaniuk, Alexander Zoufaly

**Affiliations:** 1 Department of Medicine IV, Klinik Favoriten, Vienna Healthcare Group, Kundratstraße 3, 1100 Vienna, Austria; 2 Present Address: Faculty of Medicine, Sigmund Freud University, Vienna, Austria; 3 Department of Laboratory Medicine, Klinik Favoriten, Vienna Healthcare Group, Kundratstraße 3, 1100 Vienna, Austria

**Keywords:** Infectious diseases, SARS-CoV-2

## Abstract

The causative agent of the ongoing Corona virus disease 2019 (COVID-19) pandemic, Severe acute respiratory syndrome coronavirus 2 (SARS-CoV-2), has acquired a considerable amount of mutations, leading to changes in clinical manifestations and increased transmission. Recent studies based on animal disease models and data from the general population were reporting a higher pathogenicity of the BA.2 sublineage compared to BA.1. The aim of this study was to provide real world data on patients with the SARS-CoV-2 Omicron BA.1 and BA.2 subvariants treated at our center, highlighting similarities and differences in the clinical disease course. We retrospectively collected and analyzed the data of adult patients admitted with confirmed SARS-CoV-2 infection at the Department for Infectious Diseases and Tropical Medicine, Klinik Favoriten, Vienna, Austria. Patient characteristics including age, underlying diseases, vaccination status and outcome were compared between patients with the BA.1 and BA.2 subvariants. Between January 2022 and May 2022 we included 168 patients infected with Omicron BA.1 and 100 patients with BA.2. Patients admitted with BA.2 were significantly older, more often fully immunized and required less dexamethasone than patients with BA.1. No substantial differences were identified between patients infected with BA.1 and BA.2 regarding BMI, laboratory findings, need for supplemental oxygen, mortality and other evaluated comorbidities excepting active malignancies. The significantly larger percentage of fully immunized patients admitted with BA.2 is pointing to an increased transmissibility of this subvariant, while the comparable outcome of a somewhat older and sicker patient population might be indicative of reduced virulence.

## Introduction

The ongoing COVID-19 pandemic, which is caused by the infection with the novel Severe acute respiratory syndrome coronavirus type 2 (SARS-CoV-2), was first identified as a cluster of pneumonia of unknown origin in December 2019 in the city of Wuhan, China, and spread quickly around the world becoming a global public health crisis^1–3^.

Since the beginning of the pandemic it became clear that the clinical manifestation of SARS-CoV-2 infection differs strongly between individuals, ranging from asymptomatic disease course to fulminant respiratory failure, multiple organ dysfunction and death^4–7^.

Studies conducted throughout the pandemic also reveal that the manifestation and severity of the disease has changed over time^8–13^.

SARS-CoV-2 is a positive-sense single-stranded RNA virus which exhibits, likewise to other RNA-viruses, a high rate of mutation due to their error-prone RNA-dependent RNA-polymerase^1,14–16^, leading to consequences such as changes in clinical manifestations, increased transmission and reduced potency of vaccines through immune escape^8–11,17–19^. The B.1.1.529 (Omicron) variant is countering efforts of containment especially through its' remarkable resistance to neutralization by vaccine-induced antibodies caused by changes in the receptor-binding domain (RBD)^20–23^.

Both BA.1 and BA.2 were first identified in November 2021 in Africa and their spike proteins contain 37 mutations and 31 mutations, respectively^21^, which have led to breakthrough infections^24–26^. Mutations of the spike protein that differentiate BA.2 from BA.1 are suspected to be involved in eluding recognition by neutralizing antibodies^21,27,28^, leading to a higher transmissibility^29,30^.

Recent data is to some extent contradictory in respect of the clinical manifestation of the aforementioned Omicron subvariants, which highlights the need for more clinical studies. Research conducted on animal disease models are predicting a higher pathogenicity of the BA.2 sublineage compared to BA.1^31^. The REACT-1 study, which surveys the symptomatology of the general population in England, reported an increase in flu-like symptoms and greater interference with daily life in comparison to BA.1^10^, while several other studies reported a low clinical severity of BA.2^32^ and similar outcomes to BA.1^33,34^.

Similarly to the global epidemiologic situation, Austria experienced a COVID-19 surge in the beginning of 2022, peaking at 64.038 new cases on the 17.03.2022^13,35^.

The aim of this study was to provide real world data on patients infected with the dominating SARS-CoV-2 Omicron BA.1 and BA.2 variants treated at our center during the aforementioned peak in COVID-19 cases in Austria, highlighting similarities and differences in the clinical disease course.

## Methods

We retrospectively collected and analyzed the data of adult subsequent patients admitted with confirmed SARS-CoV-2 infection at the general ward and intensive care unit of the Department for Infectious Diseases and Tropical Medicine, Klinik Favoriten, Vienna, Austria.

We included data of all consecutive patients which were RTPCR positive for BA.1 and admitted to our department between 01.01.2022 and 28.02.2022 and all consecutive patients which were RTPCR positive for BA.2 and admitted to our department between 02.02.2022 and 04.05.2022, except patients who were not discharged or deceased until the period of data collection. Data was collected between 03.03.2022 and 14.07.2022.

The presence of SARS-CoV-2 was analyzed on a Cobas 6800 system (Roche, Mannheim, Germany) using real-time polymerase chain reaction Cobas SARS-CoV-2 Qualitative assay (Roche, Mannheim, Germany) for qualitative analyses.

The PCR assay represents a dual target test, designed for the detection of SARS-CoV-2 RNA in nasopharyngeal swab samples of infected patients. Conserved regions within the ORF 1a/b and E genes are targeted to ensure high accuracy.

The SARS-CoV-2 viral variant was identified using melting curve analysis (VirSNiP SARS-CoV-2 Spike 371L 373P 452R test, TIBMolbiol, Berlin, Germany).

Routine application of test revealed high sensitivity for samples with Ct < 30.

Variant identification was based on melting characteristics specific for the mutations BA.1 and BA.2 according to the manufacturer’s instructions.

Antibody levels against the receptor-binding domain (RBD) of the SARS-CoV-2 spike protein were measured on Cobas 8000 platform (Roche, Mannheim, Germany) with the Elecsys Anti-SARS-CoV-2 S (Roche, Mannheim, Germany) assay.

This immunoassay allows in vitro quantitative determination of total antibodies to the SARS-CoV-2 S protein RBD in human serum.

The results are displayed in Roche units (U)/ml, which correlate to the WHO standard (binding antibody units (BAU)/ml). According to the manufacturer’s method sheet, samples with anti-SARS-CoV-2 S concentrations above the measuring range (0.4–250 U/mL) were diluted up to 1:10. Higher results were reported as > 2500 U/mL.

The overall specificity in the package insert of the manufacturer was 99.98%, sensitivity 98,8%, respectively.

The data regarding patient vaccination status was obtained from the Austrian national registry for COVID-19 vaccinations. Fully vaccinated patients were considered those with completed primary vaccination scheme.

Our department applies the criteria of the WHO clinical progression scale regarding to mild, moderate and severe COVID-19 cases^36^. Mild cases are considered those which are either asymptomatic or show SARS-CoV-2 typical symptoms, but do not require hospitalisation.

Moderate disease is defined as COVID-19 associated hospitalisation with, at most, the need for low-flow oxygen supplementation.

Severe cases are defined as COVID-19 associated hospitalisation with the need of high-flow oxygen suplementation, non-invasive ventilation or mechanical ventilation.

### Statistical analysis

The statistical analyses were carried out in GraphPad Prism Version 9. Categorical data was tested for significance with the Fisher's exact test. Non-categorical data was first tested for normality (D'Agostino & Pearson test, Anderson–Darling test, Shapiro–Wilk test and Kolmogorov–Smirnov test). The passing of the normality test led to a t-test, while data that wasn't normally distributed was tested with the Mann–Whitney test or, when comparing antibody levels after 0, 1, 2 and 3 vaccines, the Kruskal–Wallis Test.

### Ethical approval and consent to participate

Ethics approval from "Ethikkommission der Stadt Wien" was obtained, informed consent has been waived by the approving ethics committee. All methods were performed in accordance with relevant guidelines and regulations.

## Results

The characteristics of our patient population are summarized in Table 1.

**Table 1:** Patient characteristics.

	Omicron BA.1 (n = 168)	Omicron BA.2 (n = 100)	*P*-value
Demographic parameters			
Age (years)	65,4 (± 18,4)	70,2 (± 16,3)	0,0465
Male/Female/diverse	86(51%)/82/0	43(43%)/57/0	0,2081
Body mass index	26,6 (± 6,6)	27,80 (± 7,6)	0,4407
SARS-CoV-2 vaccination (fully vaccinated)	64/168 (38%)	59/100 (59%)	0,0010
Median antibody value (U/mL)	683 (IQR = 2500)	1335 (IQR = 2477,7)	0,1943
Active smoker	39 (23%)	15 (15%)	0,1171
Underlying disease			
Diabetes	49 (29%)	33 (33%)	0,5838
Chronic kidney disease	37 (22%)	33 (33%)	0,0613
Coronary artery disease	34 (20%)	26 (26%)	0,2914
Atrial fibrillation	30 (18%)	22 (22%)	0,4274
COPD/Asthma	28 (17%)	20 (20%)	0,5129
Arterial Hypertension	99 (59%)	58 (58%)	0,8986
Immunosuppression	20 (12%)	17 (17%)	0,2737
Active malignancy	21 (13%)	24 (24%)	0,0181
Chronic liver disease	11 (7%)	10 (10%)	0,3495
COVID-19 admission			
Ct-value on admission	21,9 (± 4,3)	23,1 (± 6,1)	0,2356
F_i_O_2_ on admission (%)	27,8 (± 16,5)	25,3 (± 11,5)	0,3868
Respiratory rate on admission	19,9 (± 5,4)	18,8 (± 4,1)	0,3443
Days since onset of symptoms	3,5 (± 4,4)	3,03 (± 4,5)	0,0318
CRP (mg/L)	55,6 (± 65,5)	58,0 (± 71,8)	0,8942
Leucocytes (G./L)	7,8 (± 4,3)	8,5 (± 7,5)	0,3058
BUN (mg/dL)	22,4 (± 17,2)	24,5 (± 18,9)	0,4786
Ferritin (μg/L)	404,7 (± 493,6)	432,4 (± 514,6)	0,6534
COVID-19 disease course			
Need for low-flow supplemental oxygen	58 (35%)	33 (33%)	0,8940
High flow supplemental oxygen	10 (6%)	7 (7%)	0,7976
Mechanical ventilation	5 (3%)	1 (1%)	0,4161
ICU stay	19 (11%)	6 (6%)	0,1934
Days until Ct-values > 30	13	13	0,4370
Duration of hospital stay	12,3	13,2	0,1777

Age, body mass index, Ct-value at admission, fraction of inspired oxygen (FiO_2_) at admission, Respiratory rate at admission, Days since onset of symptoms, CRP, Leucocytes, BUN, Ferritin are shown as mean values with standard deviation. U = Roche units = binding antibody units (BAU); Ct = cycle treshold of the polymerase chain reaction test; F_i_O_2_ = fraction of inspired oxygen; mg = milligram; L = liter; G. = Giga; dL = deciliter; μg = microgram; ICU = intensive care unit; fully vaccinated = 3 or more vaccines against SARS-CoV-2; active malignancy = diagnosed malignancy currently receiving anticancer-therapy and/or under current surveillance; days since onset = days since onset of symptoms or first positive PCR-Test in case of asymptomatic patients.

We included the data of 168 patients infected with Omicron BA.1 and 100 patients with BA.2

### Demographic parameters

Comparing the baselines characteristics, one notable difference between the two groups is the age: The patients admitted with BA.2 were, on average, 5 years older than their BA.1 counterparts. Moreover, a significantly higher percentage of patients admitted with BA.2 were fully vaccinated (59% vs. 38,09%). Accordingly, the median antibody value of BA.1 patients on admission was 683, while those infected with BA.2 presented with a median antibody value of 1335. However, this difference was not significant (*p* = 0,1943).

There was no statistically significant difference in BMIs of patients infected with BA.1 and BA.2. However, the median value of BMI in mechanically ventilated patients was 34,3, while that of patients without need for mechanical ventilation was 25,8, highlighting a significant difference (*p* = 0,0347). It is worth mentioning that only six patients were in need of mechanical ventlation.

### Underlying diseases

Patients admitted with BA.2 were burdened by active malignancies in a larger quota. The rest of the reviewed comorbidities did not show significant differences but there was a tendency of more underlying diseases in BA.2 patients (eg. chronic kidney disease 22% in BA.1 vs 33% in BA.2). The most prevalent underlying disease present in patients infected with BA.1 and BA.2 was arterial hypertension with 58,9% and 58%, (*p* = 0,8986) respectively.

### COVID-19 admission

The Ct-value, F_i_O_2_, respiratory rate and laboratory findings on admission were comparable in both groups—indicating no significant difference in CRP, BUN, leucocytes or ferritin.

### COVID-19 disease course

Among all patients infected with BA.1, 36% were administered dexamethasone, while only 23% BA.2 patients seemed to be needing a glucocorticoid therapy (*p* = 0,0397). Our Standard-of-Practice (SOP) recommends dexamethasone treatment in patients with need for supplemental oxygen caused by COVID-19 disease. We consider a dexamethasone treatment to be a better indicator for a moderate/severe COVID-19 disease course than the need for oxygen supplementation, as the latter had more confounding variables.

There was no significant difference in the ratio of BA.1 and BA.2 patients who received antiviral therapy (64% vs. 75%, respectively, *p* = 0,0773), Sotrovimab (33% vs. 27%, *p* = 0,3391) or immunomodulatory therapy (9% vs. 3%, *p* = 0,0765).

### Need for supplemental oxygen and Ct-values

**Figure 1:**
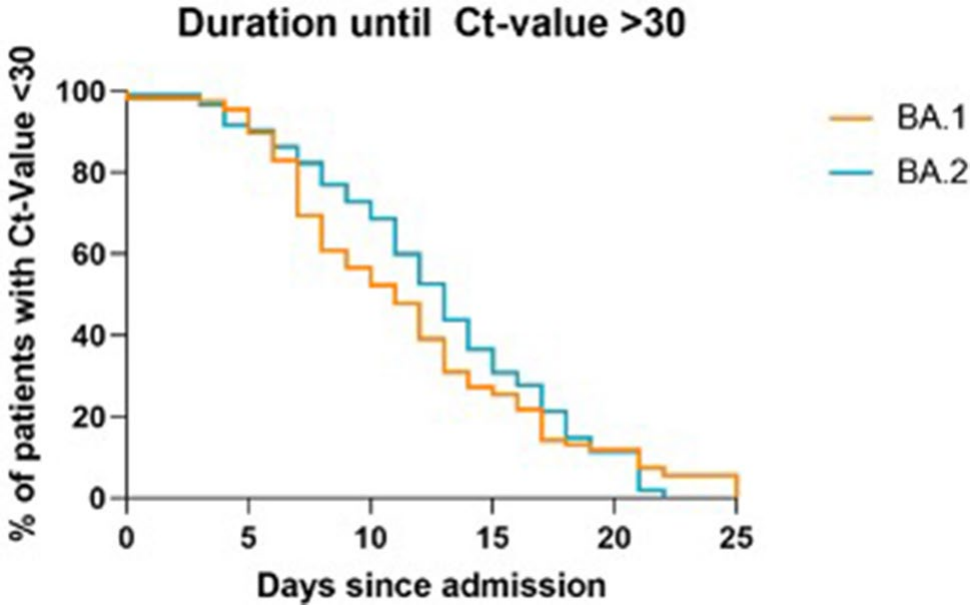
Kaplan–Meier plot showing the percentages of BA.1 and BA.2 patients with Ct values under 30 during in-hospital stay. There was no significant difference in the two curves using the logrank (Mantel-Cox) test.

There were no significant differences in Ct-value upon admission and during the in-hospital stay (Fig. 1), FiO_2_ and respiratory rate on admission, but significant difference in days between onset and hospital admission, highlighting a shorter duration for patients infected with BA.2.

**Figure 2:**
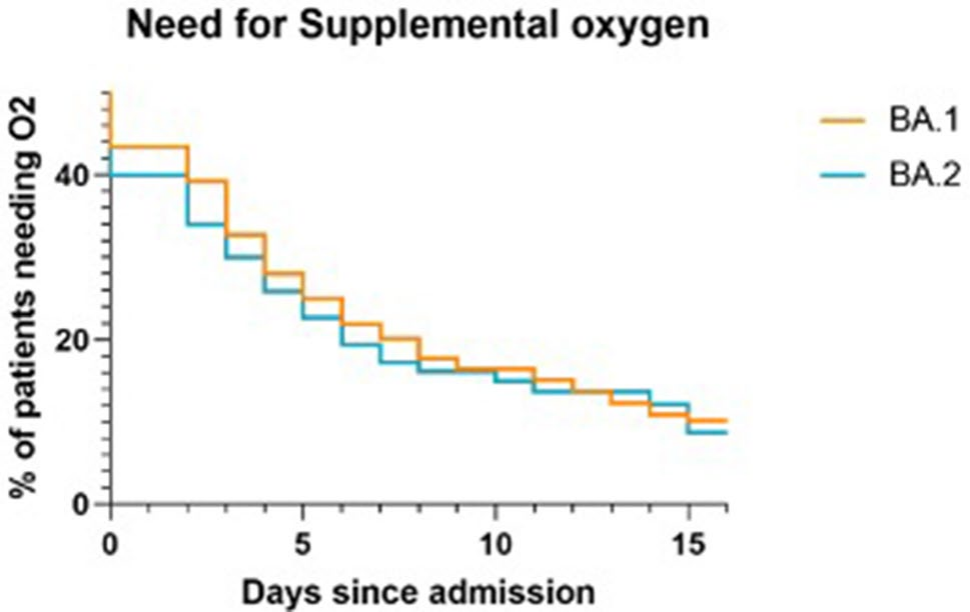
Kaplan–Meier plot showing the percentages of BA.1 and BA.2 patients needing supplemental oxygen during their in-hospital stay. There was no significant difference in the two curves using the logrank (Mantel-Cox) test.

The proportions of patients needing supplemental oxygen in the form of low-flow, high-flow oxygen or mechanical ventilation (Fig. 2), as well as an ICU stay were similar in both groups. The duration of hospital stay and elapsed days until Ct-values surpassed 30 were also comparable (Fig. 1). Unsurprisingly, the mortality of the two groups was also alike: 13 (8%) BA.1 and 11 (11%) BA.2 patients died during their hospital stay.

The antibody concentration did have an effect on the need for supplemental oxygen in both sublineages: lower antibody levels were associated with more need for supplemental oxygen. BA.1 patients needing supplemental oxygen had a median antibody value of 157,5 U/mL, while those who did not require it had a median antibody value of 1128 U/mL (*p* = 0,0123). BA.2 patients had similar differences, with 539 U/mL in the case of those who required supplemental oxygen and 1992 U/mL in the case of those who did not (*p* = 0,0489). The number of vaccines was directly proportional with the concentration of antibodies in our patients (*p* < 0,0001).

## Discussion

### Transmissibility

At our department, BA.1 was most prevalent during the months of January 2022–late February 2022, coinciding with a spike of COVID-19 cases in the general population of Austria with a maximum of 40.419 new cases on the 10.02.2022. Immediately followed by this wave came another with even higher numbers of daily new infections, peaking at 64.038 new cases on the 17.03.2022, and coinciding with the predominance of BA.2 at our department^13,35^. This epidemiological data is consistent with the predictions and findings of other studies which mention a higher transmissibility of BA.2 in comparison to BA.1^21,29,30^.

### Virulence

It is crucial to distinguish between patients who were hospitalized primarily because of COVID-19 and received treatment for it, and those who were hospitalized for a different reason and transferred to our department solely for isolation purposes.

This study evaluated the need for supplemental oxygen and COVID-specific treatment according to our SOP. As less than half of our patients (43% of BA.1 patients and 41% of BA.2 patients) needed supplemental oxygen (Fig. 2), it could be assumed that only a fraction of the admissions were primary COVID-19 cases. Likewise, one previous study, which was conducted in a medical center in the Netherlands between December 2021 and February 2022, found that only 45% of the adult patients admitted with the SARS-CoV-2 Omicron variant were primary COVID-19 cases^37^.

Interestingly, a significantly higher proportion of BA.1 patients received dexamethasone. This presumably indicated that a significantly higher percentage of BA.1 patients needed glucocorticoids for a COVID-19-associated hyperinflammatory syndrome as compared to patients with BA.2.

There was no significant difference between those infected with BA.1 and BA.2 with regard to the degree of oxygen supplementation. This could be indicative of a generally mild clinical manifestation considering the high age of BA.2 patients. However, our small sample size might have diluted a difference between groups, as the average FiO_2_ was numerically lower in BA.2 patients. In addition, higher antibody titers had a protective character against fulminant disease with a need for oxygen therapy in both sublineages. As the number of vaccines correlated with the concentration of antibodies in our patients, this finding was consistent with previous data which points to booster vaccines positively affecting the clinical course, despite infection with newer variants which have a notable ability for immune escape and for causing breakthrough infections^38,39^.

There was no statistically significant difference in BMIs of patients admitted with BA.1 and BA.2. This notwithstanding, it is important to mention that those in need of mechanical ventilation had a significantly higher BMI (*p* = 0,0347), supporting existing evidence^40,41^ and confirming that this remains a risk factor in the case of infections with BA.1 and BA.2 as well.

The results of our study confirm previous research which indicates a higher transmissibility of BA.2^29^, as it can escape vaccine-induced antibodies and can lead to hospitalization in fully immunized patients in a larger proportion than BA.1 (*p* = 0,001). Contrary to previous evidence based on infections in the general population^10^ and predictions derived from animal models^31^ which report a stronger symptomatology and increased disease severity of BA.2, our findings present a change in the demographics of patients hospitalized with BA.2, such as older age and a larger proportion of patients with active malignancies, but a similar outcome to BA.1 (need for oxygen, ICU stay, respiratory rate on admission, laboratory findings), suggesting a somewhat lower virulence of BA.2. The higher age at admission in the case of BA.2 may also be indicative of reduced need for hospitalization in younger patients, or of increased community spread in older patients.

This study has a number of limitations. As a retrospective single center study with a rather low sample size, there is a high susceptibility to bias. Furthermore, we included the data of only 100 BA.2 patients, which might have been too little to unveil greater differences between the two subvariants.

Another limitation is the early discharge of patients with mild disease or rapid improvement of their clinical status—these patients were administered antiviral therapy and antibodies and sometimes discharged while still infectious. As a consequence, we lack information about the necessary time to clear the virus in patients with mild clinical manifestations.

Exact FiO_2_ values needed during the first 15 days of stay at our department were thoroughly documented. Nevertheless, some values are missing due to patients being discharged, transferred, moribund patients not tolerating high-flow oxygen supplementation or in case of death. This could have led to a bias regarding the cumulative FiO_2_ values of the later days.

To summarize our evidence, the behavior of SARS-CoV-2 might take a natural evolutionary course. Similar to many other viruses, the mutations that provide a growth advantage and increased fitness prevail. As the newer variants of SARS-CoV-2 show higher transmissibility by escaping neutralizing antibodies, they might also cause less severe illness, hopefully leading in a more distant future to the possibility of a normalized coexistence with this pathogen.

## Data Availability

The datasets used and/or analysed during the current study are available from the corresponding author on reasonable request.
